# Some Notes on Arrow-Wounds

**Published:** 1866-06

**Authors:** Elliott Coues

**Affiliations:** U.S.A.


### SOME NOTES ON ARROW-WOUNDS.

BY ELLIOTT COUES, M.D., U.S.A.

During a residence of more than a year, in the interior of Arizona Territory, as surgeon of a United States Post, (Fort Whipple,) at a time when both our civil and military force was in incessant warfare with the hostile Apaché Indians, the writer had numerous cases of arrow-wounds under his care, in private practice as well as in the line of official duty. As reports of injuries of this sort are by no means often brought into general notice, to the following desultory notes of wounds produced by the peculiar stone-headed arrows of the Indians just named, some little interest may possibly attach.*

The Apaché arrow consists of a light, tough reed, nearly or quite three feet long, at one end tri-feathered and slotted for the bow-string in the usual manner, and painted with some emblematical red and blue waved lines. Into the distal extremity is inserted a short and slender, but stout piece of firm wood, some eight or ten inches long, hardened and straightened in the fire, and thickly besmeared with a black, gummy substance.

The head is apparently a small and trifling affair, compared with the results it is capable of producing. It is made from some species of quartz, chalcedony, obsidion, etc., and is always either white or black in color. It is an inch or somewhat less in length, by about a-third of an inch in greatest width; in shape a narrow isosceles triangle. It is quite uniform in size and shape. I think I never saw one much over the dimensions stated. It is very thin, and its apex is chipped to an extremely fine point. Its sides have usually three, sometimes only one or two jagged notches near the base. Its bulbs are generally long and sharp. The base is notched where it rests on the shaft. In the end of the hardwood shaft, just described, is a slight notch, actually not so deep as that which receives the bow-string; on which is dropped a little very tenacious gum; and then the stone-head is lightly pressed into place. There is no projecting handle for insertion into the wood. No thongs or wrapping of any sort are used; and so frail is the connection between the head and shaft, that the Indians themselves are obliged to carry their arrows with great care.

The bow is a large, strong one, four to six feet long; almost straight to near either end, where it is abruptly much curved.

It will be seen that this weapon, the injuries from which we are to notice, is quite a different affair from the short, stout, heavy arrow, with a large and long triangular, or somewhat diamond-shaped soft metal head, bound by sinews to the shaft, used by the mounted Indians of the plains; although they agree

* For a very elaborate memoir on arrow-wounds, see Dr. G. H. Bill, U.S.A., in the *Amer. Journal Med. Sci.*, for Oct., 1862, p. 365; and compare nature of wounds by the strong, large, metal heads of the Navajos with those indicated in this paper.

in the respect, that both make very ugly wounds. I do not think that the penetrative force of the Apaché arrow equals that of the Comanché or Navajo. The characteristics of the Apaché arrow-head are essentially these: 1, its minute size; 2, its jagged edges and angles; 3, its extreme friability; 4, its very ready separation from the shaft; 5, its probable poisoning in some instances.

CASE. Illustrating consequences of minute size of arrow-head. Arrow horizontally entered outer aspect of thigh of a large muscular man, its wide axis parallel with fibres of m. vastus ext., severed profunda or one of its larger branches; hæmorrhage to fainting, and repeated. Saw patient three hours from reception of injury; after he had ridden, supported on either side, for five miles. He had pulled out the shaft immediately upon being struck. Very moderate swelling or increased heat of limb. Orifice of entrance a simple slit, 3-10ths of an inch long. By no amount of delicacy and patience in the manipulation of a small silver probe, with the limb in various positions, could the former be introduced more than about an inch. Head consequently undetectable, and therefore unextractible. Expectant treatment necessitated; nothing special to be done beyond ordinary constitutional care. The tumefaction rapidly abated; and the wound closed completely in a few days. No abscesses, or unusual amount of irritation from presence of stone; but the limb was weak and practically useless for several weeks. Two months afterward, the patient could *feel* the head on the inside of the thigh, close by the femoral; and motion, especially horse-back exercise, increased the vividness of his intelligent impression that there it was; although it was not to be detected by surgical palpation. It has already, or doubtless will be hereafter extracted by cutting in a manner not widely dissimilar from that for the operation of tying the femoral near the apex of Scarpa's triangle. This case is so well expressive of the essential characters of several others that fell to my lot, that they need not be here detailed. I soon learned to regard the innocent-looking little slit with anxious doubts of its directing me to the cause of it, when I saw it parallel with muscular fibres.

The above described kind of injury is evidently as near a "punctured" wound in essential characteristics, as can well be made by any edged weapon.

The jagged edges and sharp angles of the head, do not seem to be, at least in their affects upon muscular tissue, as pernicious as they are malevolent in appearance. They certainly must decrease rather than aid the penetrative force or the arrow; and the amount of lacreation they are capable of producing is by no means a serious complication. I have thought indeed in some instances, that this degree of lacreation was the reverse of deprecable; affording more facile entrance to the probe, and afterward more ready exit to the inevitable pus.

At the same time the effect of these notches and angles is of importance with reference to the chances of the encysting of heads which cannot be traced and extracted. As might be expected, it is exceedingly rare that these sharply triangular and jagged bits of stone excite so little disturbance that the encysting process can be accomplished. This can only take place under some peculiarly favorable circumstances, as to position, etc. The irritation is almost invariably sufficient to excite suppuration; consequent abscesses, (unless the wound is very open;) and thus a gradual working of the head toward the surface; this "pointing" being often at a considerable distance from the place where it might have been expected. It is only rarely, I think that the head works out, *retrorsum*, along the track of its entrance.

The extreme friability of the head produces results which must be taken into consideration, as one of the most common and troublesome features of the wound. When the head impacts on bone—and it generally traverses soft tissue till halted in this way—the chances of its shivering into bits vastly preponderate over the probability of its becoming fixed or glancing.

Case. Arrow-head struck under edge of malar, rasped off some periosteum, shivered as it glanced downward, and over twenty minute fragments, (in addition to two or three large ones extracted,) came away gradually from the fat and muscle lying under this bone.

Case. Arrow-head struck transverse process of eleventh dorsal vertebra; shivered; fortunately without glancing into the thorax. Three large pieces extracted immediately. Numerous smaller fragments came away in the suppuration.

Case. Arrow-head struck inferior angle of scapula. A large piece was at once extracted, which represented the whole of the head except about a fourth of an inch of its apex, which was firmly fixed in the bone. As this bit would not have been extractible except by much cutting, and then prying and wrenching, I somewhat hesitatingly allowed it to remain; possibly an injudicious procedure, although warranted by the result. The wound closed completely in about two weeks, and there were not, at least during the several subsequent months that I was with the patient, any unfavorable symptoms.

The above cases could be multiplied, but are sufficient for my present purpose. It will be evident that we need not be afraid of a little judicious audacity in the division of muscular tissue, while cutting a way for our forceps, since the enlarged orifice is favorable, if not indeed necessary, for the ready discharge of pus as well as of quartz débris. This same indication, so important to be fulfilled must also govern all subsequent dressing of the wound. *Dress openly;* better not dress at all, than occulade the orifice. Particularly be careful that subjacent tissue is sound, before allowing integument to reunite.

Occasionally an arrow-head will neither shiver nor impact; but on striking bone in an exposed situation, *e.g.*, tibia, ulna, etc., will rebound with great force. CASE. Head struck middle of outer aspect of ulna, and rebounded. Resulting injury more of an ulcer than anything else. A scab rapidly formed; seeing which I merely protected it from friction by the clothes, and it "got well itself" directly.

So frail is the connection between the head and the shaft, that in all my little experience, I never saw or heard of an instance in which the former was removed on pulling out the latter. I do not see very well how it can occur, provided the head be buried beyond its barbs. For the matter of that, as the shaft produces ordinarily next to nothing of the sum total of injury, we may regard the missile as practically consisting of the head alone. The shaft is almost invariably seized and jerked hurriedly out by the patient at the moment of being struck. The not the less comical because painful sight of a man thus porcupinized rarely falls to the surgeon's lot, unless he is verdant or unlucky enough to get mixed up in a bush fight himself. But we must receive with great caution all opinions expressed by the patient, to the effect of his having withdrawn the head or even the whole of the shaft; for in the flurry of the moment, he does not examine what he so hurriedly pulls out; and the head he found in his clothes and presents triumphantly in proof was just as likely detached from some other arrow, which had not force to penetrate further. Again, this frantic wrenching at the stick may break off a portion of it inside. We cannot examine the wound too thoroughly before making a diagnosis. Let the following most interesting, and decidedly most mortifying case, which occurred in my private practice, exemplify these remarks.

CASE. J. Blank, young and vigorous and moderately muscular subject. Arrow entered muscles on side of back of neck, and coursed downward and forward. The head was plainly to be felt just below the clavicle of the same side. The patient stated he had pulled out the shaft immediately on being struck, some twelve hours previously. Only experienced pain when my finger pressed on the head. Feeling the latter so plainly, in front, under the clavicle, and apparently only about half an inch deep, it by no means occurred to me to stick a probe into the wound some six inches or more, for the sake of feeling it from behind; but I at once prepared to extract the head. Seated on a stump, and my patient on a barrel in front of me, the heat and flies intolerable, and only a soldier to assist, I cut carefully down upon the head by an incision parallel with the under edge of the clavicle. I found it lying on the first rib, *between it and the subclavian artery*, which latter had been somewhat shoved aside, but luckily quite uninjured. The artery was directly under my finger, and I think I could have tied it in the last portion of its course, by slightly enlarging upon the operation. I extracted about three-fourths of the head in one large piece, and afterward nearly all the rest in another smaller fragment; a little bit, however, was left in, as I could not readily find it, and no one cares particularly to poke about with a probe in the supra-clavicular triangle. A great deal of pus had followed my reaching the head with a knife; the axilla was padded and strapped tightly in view of possible abscesses; and the wound dressed openly, trusting that the fragment would come out with the suppuration; as indeed was shortly afterward the case.

But still the cut I had made continued to discharge inordinate quantities of pus. The original wound meanwhile quite healed; while the cut whence the head was extracted assumed gradually the characters of a fistula and showed no signs of closing. The patient became weak and emaciated, with considerable hectic fever, though at no time confined to his bed. Serious and distressing pulmonary symptoms had come on, chiefly indicating abscess in the chest. The patient not being under my charge except for a few days immediately after the operation, I only saw him occasionally and accidentally, and therefore cannot give more detailed notes than the above. Meeting him one day, I was surprised to learn that a piece of the shaft of the arrow, about three inches in length, had been extracted from his chest, far into which it had gradually worked. At the time I operated, it must have been lying in the muscles of the side of the neck. Simple introduction of the probe from behind would have revealed its presence and saved the poor fellow much suffering, and myself much mortification; as it could have been drawn out with the utmost ease then, had I had an idea of its presence.

"Do the Apachés poison their arrows?" is a question often asked me; and it is currently reported that they dip the heads in a deer's liver, after forcing a rattlesnake to bite it, and then allowing it to putrefy. I reply most unhesitatingly that as a general rule they *do not*, no other than the consequences of mechanical violence following in the vast majority of instances. Still I cannot affirm that such is never the case; witness the following case, in which it is difficult to account for death except upon the supposition that some toxic agent complicated a simple wound.

CASE. Private G. S., Co. F. 5th U.S. Inf. Arrow-head entered the muscles of the back, just over the lower part of the shoulder-blade; passed very obliquely upward and a little inward, chipping a bit off the scapular spine. The orifice of entrance was very small, and rounder and more jagged than usual. A probe passed to its full length readily enough, but no foreign body could be felt by it. The man was positive that the head had come out when he drew the shaft; a statement, however, to be regarded as very problematical. He complained most of pain in the muscles of the side of the neck, which was swollen and stiff in about the locality to which I had been able to pass the probe. No suspicious substance could be detected after careful palpation; and a simple expectant plan of treatment was forced upon me. The wound soon began to suppurate profusely; the discharge at first tolerably "laudable," but quickly changed to a thin, ichorous, reddish-brown fluid; mixed with which was a considerable quantity of almost pure, very dard, clotted blood; and later, shreds or masses of apparently disintegrated tissue. The orifice was enlarged; and every precaution taken to facilitate discharge and prevent abscesses; nevertheless, they began to form rapidly, and at points down the back more and more remote from the original seat of the injury. These were evacuated as soon as detected. The patient began to rapidly sink under the profuse discharge; anorexia, insomnia, vomiting, diarrhœa, and an excessively irritable and desponding state of mind, successively made their appearance; in the latter stages there was considerable delirium; and finally, in spite of all that could be done for him, he died comatose, about three weeks after receiving the injury.

Autopsy revealed that all the muscles of that side, from the occupit nearly to the hip, were in a state of disintegration; dark-colored, soft in consistency, looking in fact much like decaying liver, and having and exceedingly offensive odor. The arrow-head could not be found, after a most diligent search. It had not penetrated the thorax, nor yet had it worked into the abdominal cavity; for the parieties of both were intact. To this day the exact course of the arrow, and why it should have produced such anomalous results remain a mystery to me. But I can affirm positively that the injury which proved fatal was confined to the muscles of the back; and the sum total of its immediate effect that described above.

The constitutional disturbances following these arrow-wounds, even when the injury is confined to bone or muscle, are liable to be out of all proportion to the apparent amount of damage done. There is almost always considerable febrile action, more or less complete anorexia and sleeplessness, and derangement of all the secretions. To these may be added great irritability and intolerance of a moderate degree of pain, much dejection of spirits, haggard and anxious countenance, etc. The tendency to despondence becomes frequently a prominent symptom to be combatted, and everything should be done to cheer the patient.

I would in conclusion offer a few simple rules for the treatment of this kind of injury:—

1. Explore the wound thoroughly, even should much division of muscular tissue be thereby necessitated.

2. Extract the last fragment that can by any means be laid hold of, and cleanse the wound thoroughly.

3. Dress very lightly and openly, facilitating by every means the ready discharge of pus; watching for burrowing and abscesses, and evacuating them as soon as detected.

Attend promptly and vigorously to constitutional symptoms, combatting them by the ordinary well-known means.—*Phila. Med. & Surg. Reporter.*

